# Correlation of Clinical Frailty Scale and Outcomes in Patients Treated With Non-invasive Ventilation: A Retrospective Cohort Study

**DOI:** 10.7759/cureus.95641

**Published:** 2025-10-29

**Authors:** Ritabrata Bandopadhyay, Nirish Vaidya, Aimen Ayaz, Jasmin Maghamifar, Areefa Momtaz, Mainul Huda, Ziaudeen Ansari

**Affiliations:** 1 Internal Medicine, Sandwell and West Birmingham Hospitals NHS Trust, Birmingham, GBR; 2 Pulmonology, Sandwell and West Birmingham Hospitals NHS Trust, Birmingham, GBR; 3 Respiratory Medicine, Sandwell and West Birmingham Hospitals NHS Trust, Birmingham, GBR; 4 General Internal Medicine, Sandwell and West Birmingham Hospitals NHS Trust, Birmingham, GBR; 5 Geriatrics, Sandwell and West Birmingham Hospitals NHS Trust, Birmingham, GBR; 6 Acute Medicine, Sandwell and West Birmingham Hospitals NHS Trust, Birmingham, GBR

**Keywords:** chronic obstructive pulmonary disease (copd), clinical frailty scale, disease mortality, hypercapnic respiratory failure, non-invasive ventilation (niv), retrospective cohort

## Abstract

Background

Frailty is defined as a state of vulnerability to stressors due to physiological decline. While frailty is an increasingly recognized determinant of outcomes in acute illness, its specific role in patients receiving non-invasive ventilation (NIV) for acute type 2 respiratory failure (T2RF) remains poorly defined.

Methods

We conducted a retrospective cohort study of 112 patients treated with NIV at a district general hospital in the United Kingdom between December 2024 and April 2025. Frailty was assessed using the Clinical Frailty Scale (CFS) and categorised as CFS 1-3 (very fit to managing well), CFS 4-6 (mild to moderate frailty), and CFS 7-9 (severe frailty to terminally ill). Outcomes included inpatient mortality and readmissions within four months. Associations were analysed using the Chi-square test.

Results

Frailty was highly prevalent, affecting 90 (80.4%) patients in this cohort. Mortality rose significantly with frailty: one (4.5%) patient in CFS 1-3 group, 12 (21.4%) in CFS 4-6 group, and 14 (41.2%) in CFS 7-9 group (p = 0.006). Among survivors, 35 (41.2%) patients were re-admitted within four months, and did not differ significantly across frailty groups (p=0.400).

Conclusion

Frailty, as measured by the CFS, was a strong predictor of short-term mortality in patients receiving NIV for acute type 2 respiratory failure, independent of age. Routine frailty assessment could improve risk stratification and guide clinical decision-making in this patient group.

## Introduction

Respiratory failure is defined as a condition where there is failure of the respiratory system in performing gas exchange, namely oxygenating blood and eliminating carbon dioxide [1]. It can be classified as hypoxic (type 1) respiratory failure and hypercapnic (type 2) respiratory failure. Acute hypercapnic respiratory failure is usually caused by defects in the central nervous system, impairment of neuromuscular transmission, mechanical defects of the ribcage, and fatigue of the respiratory muscles [1].

Type 2 respiratory failure is treated with the use of non-invasive ventilation (NIV) or invasive mechanical ventilation as the main supportive measures while also treating the underlying cause [1]. NIV has been proven to be more successful in patients with hypercapnic respiratory failure in chronic obstructive pulmonary disease (COPD), but not much data exists to show its evidence of success of non-COPD conditions, with only one study showing that non-COPD patients are likely to have greater failure rates with NIV [2,3]. NIV failure and mortality are not influenced by individual factors like age and BMI, but by a combination of multiple factors together [4]. Scoring systems like Acute Physiology and Chronic Health Evaluation II (APACHE II) that consider multiple factors like age, immune status, blood pressure, heart rate, respiratory rate, white cell count, temperature, electrolytes, and renal function, along with some independent factors like serum albumin, can predict an overall mortality rate in acute type 2 respiratory failure [5].

Frailty is theoretically defined as a clinical state of increased vulnerability to stressors due to a cumulative decline in physiological systems, largely because of age. For practical assessment, it is frequently defined by a phenotypic model, where an individual meeting three out of the following five criteria, low grip strength, low energy, slowed walking speed, low physical activity, or unintentional weight loss, is considered frail [6]. Frailty is increasingly recognised as a key determinant of outcomes in hospitalised patients as seen in different studies [7]. Increasing age is not always related to functional deficits and Clinical Frailty Score is used as a measure of biological deficits and functional reserve irrespective of age [7,8].

Evidence regarding the association between frailty and mortality in patients receiving NIV for acute type 2 respiratory failure remains limited. Previous studies have largely focused on age, comorbidity, and disease severity as prognostic markers, with limited exploration of how frailty may influence both immediate and post-discharge outcomes [9,10]. A clearer understanding of this association may improve patient risk stratification, guide decisions regarding treatment escalation, and aid in advance care planning. Furthermore, we also performed this study to evaluate the relationship between frailty scores and immediate mortality outcomes in patients with acute type 2 respiratory failure.

## Materials and methods

This was a retrospective cohort study conducted at the Sandwell and West Birmingham Hospitals NHS Trust in the United Kingdom. This retrospective cohort study examined the association between frailty, measured by the CFS, and outcomes in patients treated with NIV for acute type 2 respiratory failure. The study assessed inpatient mortality and four-month readmission to evaluate how frailty influences both intermediate and short-term outcomes and guides clinical decision-making.

Study population

A total of 112 adult patients admitted to the Trust between December 2024 and April 2025 were included in the study. 

Inclusion Criteria

All patients aged 18 years or older, admitted with acute decompensated type 2 respiratory failure requiring NIV, discharged from hospital at least four months before the study period, and considered unfit for invasive ventilation or unsuitable for intensive treatment unit (ITU) admission were included.

Exclusion Criteria

Patients with acute type 2 respiratory failure managed successfully with medical therapy alone without NIV, those in whom NIV was not initiated by decision, and those who died before NIV initiation were excluded.

Data collection

Patient lists were obtained from the Respiratory Medicine department, and information was extracted retrospectively from medical records. Data were collected on the patient’s frailty status, admission outcome, and four-month re-admission to the hospital.

Frailty assessment was performed using the Rockwood CFS [11] at the time of admission (Table 1). The CFS is a validated 9-point scale widely used in clinical research and practice. Patients were grouped as: very fit to managing well (CFS 1-3), mild to moderate frailty (CFS 4-6), and severe frailty to terminally ill (CFS 7-9). Only descriptive categorisation was used in this study; no reproduction of the original scale figure or chart has been included.

**Table 1:** Rockwood Clinical Frailty Scale Reference: Rockwood et al., 2005 [11]

Score	Description
1: Very Fit	People who are robust, active, energetic, and motivated. These people commonly exercise regularly and are among the fittest for their age.
2: Well	People who have no active disease symptoms but are less fit than those in category 1. Often, they exercise or are very active occasionally, e.g. seasonally.
3: Managing Well	People whose medical problems are well controlled but are not regularly active beyond routine walking.
4: Vulnerable	While not dependent on others for daily help, often symptoms limit activities. A common complaint is being “slowed up” or being tired during the day.
5: Mildly Frail	These people often have more evident slowing and need help in higher-order IADLs (finances, transport, heavy housework, medications).
6: Moderately Frail	People who need help with all outside activities and with keeping house. Inside, they often have problems with stairs and need help with bathing and minimal assistance with dressing.
7: Severely Frail	Completely dependent for personal care, from whatever cause (physical or cognitive). Even so, they seem stable and not at high risk of dying within 6 months.
8: Very Severely Frail	Completely dependent, approaching the end of life. Typically, they could not recover even from a minor illness.
9: Terminally Ill	Approaching the end of life. Life expectancy <6 months, but not otherwise evidently frail.

Outcomes of the initial admission (discharged home or deceased due to respiratory cause) were recorded, as well as any readmissions with acute type 2 respiratory failure requiring NIV within four months among survivors.

Data were collected retrospectively from electronic health records of patients treated with NIV during the study period. All data were anonymised prior to analysis and cross-verified to ensure accuracy and completeness. Missing data were excluded from analysis where appropriate.

Statistical analysis

Data were analysed using descriptive statistics to summarise baseline demographic and clinical variables. Categorical variables are presented as frequencies and percentages. Associations between frailty categories, inpatient mortality, and four-month readmission were assessed using the Chi-square test of independence. A p-value of <0.05 was considered statistically significant at the 5% level of significance. All analyses were performed using IBM SPSS Statistics for Windows, Version 28.0 (IBM Corp., Armonk, New York, United States). No missing data required imputation.

## Results

A total of 112 patients were included in the study. COPD was the most common diagnosis, present in 72 (64%) patients, followed by 19 (16.9%) patients with obesity hypoventilation syndrome (OHS). Patients had overlapping conditions such as COPD with heart failure or OHS, neuromuscular disorders including motor neurone disease, and isolated heart failure. This distribution reflects the heterogeneous patient population admitted with acute decompensated type 2 respiratory failure requiring NIV.

Frailty status, assessed using the Rockwood CFS, demonstrated that most patients were living with some degree of frailty at admission. Only 22 (19.6%) patients were classified as ‘very fit to managing well’, while 56 (50.0%) were categorised as ‘mild to moderate frailty’ and 34 (30.4%) as ‘severe frailty to terminally ill’ (Table 2).

**Table 2:** Frailty score among population (N=112)

Frailty category	Number	Percentage
Very fit to managing well	22	19.6
Mild to moderate frailty	56	50.0
Severe frailty to terminally ill	34	30.4

The CFS scores among the study population ranged from 1 to 9, with a median (Q2) of 5. The interquartile range of 3 indicates moderate variability in frailty among patients treated with non-invasive ventilation (Table 3).

**Table 3:** Distribution of Clinical Frailty Scale (CFS) scores among study participants

Statistics	CFS Value	Interquartile range (IQR)
Q1	4	3
Q2	5	
Q3	7	

Overall mortality during admission was 27 (24.1%), with 85 (75.9%) patients surviving (Table 4).

**Table 4:** Mortality among the patients (N=112)

Mortality	Frequency	Percentage
Deceased	27	24.1
Alive	85	75.9

Mortality rates rose progressively with increasing frailty. Among patients who were in ‘very fit to managing well’ category, only one (4.5%) had a fatal outcome, compared with 12 (21.4%) in the ‘mild to moderate’ frailty group and 14 (41.2%) in the ‘severe frailty to terminally ill’ group. Statistical analysis using the Chi-square test confirmed that this association was statistically significant (χ² = 10.236, p = 0.006), highlighting frailty as a strong determinant of short-term mortality in this cohort (Table 5).

**Table 5:** Association of frailty categories with mortality (N=112)

Frailty categories	Mortality, n (%)	Alive, n (%)	Chi-square value	p value
Very fit to managing well	1 (4.5%)	21 (95.5%)	10.236	0.006
Mild to moderate frailty	12 (21.4%)	44 (78.6%)		
Severe frailty to terminally ill	14 (41.2%)	20 (58.8%)		

Among the 85 survivors, 35 (41.2%) patients experienced at least one re-admission within four months of discharge from the hospital, while the remaining 50 (58.8%) patients did not have any re-admissions (Table 6).

**Table 6:** Re-admissions within four months among the survisors (N=85)

Characteristics	Frequency	Percentage
Re-admission(s)	35	41.2
No Re-admission(s)	50	58.8

Re-admission rates showed a similar upward trend across frailty groups, with six (28.6%) patients in ‘very fit to managing well’ group having re-admission within four months of discharge, compared with 20 (45.5%) in the ‘mild to moderate frailty’ group and 9 (45.0%) in the ‘severe frailty to terminally ill’ group. Despite this pattern, the differences were not statistically significant (χ² = 1.831, p = 0.400) (Table 7).

**Table 7:** Association of Frailty categories with re-admission (N=85)

Frailty categories	No Re-admission, n (%)	Re-admission, n (%)	Chi-square value	p value
Very fit to managing well	15 (71.4%)	6 (28.6%)	1.831	0.400
Mild to moderate frailty	24 (54.5%)	20 (45.5%)		
Severe frailty to terminally ill	11 (55%)	9 (45%)		

## Discussion

According to the WHO, COPD is the fourth leading cause of death worldwide, causing 3.5 million deaths in 2021, approximately 5% of all global deaths, and is the eighth leading cause of poor health worldwide [12]. Patients with COPD have a higher mortality compared with the general population, and it is a common health problem in the United Kingdom involving a substantial proportion of individuals [13]. Hypercapnic respiratory failure, a state of reduced alveolar ventilation with respiratory acidosis, is often caused by common respiratory diseases such as COPD, OHS, and COPD overlap syndrome with OHS [14]. Hypercapnic respiratory failure is a frequent cause of admission and a common cause of mortality in many patients, particularly those with COPD [15]. Hence, it is very important to understand the various risk factors affecting mortality rates in these patients.

NIV remains a cornerstone of management for acute type 2 respiratory failure, particularly in the context of COPD exacerbations, where its benefits in reducing mortality and the need for intubation are well established [2,3,16]. However, outcomes are more variable in non-COPD populations, with several studies reporting higher failure rates in patients with alternative causes of respiratory failure [2]. Overall, patients with hypercapnic respiratory failure have been found to have a high risk of 30-day readmission and death [15]. The present study focused exclusively on patients who were deemed unsuitable for invasive mechanical ventilation, as they represent the group in which NIV is most commonly used in clinical practice. While this selection criterion may limit generalisability to all patients receiving NIV, it reflects real-world clinical decision-making in acute settings and thus provides valuable insights into outcomes within this specific population.

Frailty has emerged as a potential determinant of clinical outcomes in acute illness [8]. The CFS offers a pragmatic and reproducible measure of biological rather than chronological age, reflecting functional reserve and vulnerability to stressors [5,7]. Studies have repeatedly shown that the outcome of patients with COPD requiring NIV did not depend on their chronological age alone [2,9,17]. However, higher CFS has been shown to predict more adverse outcomes in patients admitted to the hospital, irrespective of the health condition, in multiple studies [18-20]. Limited studies have examined the relationship between CFS and respiratory conditions, particularly in non-COPD populations. However, available evidence indicates that frailty is common among older people with COPD, is associated with a decline in lung function [21], and is linked to increased rates of exacerbations and mortality [22,23]. 

In our study, frailty emerged as a strong predictor of short-term mortality in patients requiring NIV for acute type 2 respiratory failure. Mortality rose progressively with increasing CFS categories, and this association was statistically significant (p = 0.006). These findings align with a previous study done in the United Kingdom by Hatton et al., which also demonstrated a significant relationship of frailty with mortality in patients receiving acute NIV [24]. Similarly, a study by Kara et al. involving 103 patients admitted to the intensive care unit (ICU) found higher rates of NIV failure in frail individuals, supporting the prognostic importance of frailty [25]. Collectively, these results suggest that the CFS can be an independent predictor of short-term mortality in patients receiving NIV and could be a valuable tool to guide clinical decision-making. Although treating CFS as a continuous variable could potentially provide additional granularity, categorical grouping was chosen to facilitate clinical interpretation and align with previous studies.

While previous literature has shown that frailty is an independent risk factor for hospital readmission [26,27], our findings did not demonstrate a statistically significant relationship between frailty and four-month readmission rates (p = 0.400). Although there was a trend toward higher readmission rates with increasing frailty, this did not reach statistical significance, possibly due to sample size limitations or confounding by comorbid conditions. Larger multicentre studies may be needed to clarify the role of frailty in predicting readmissions in patient groups treated with acute NIV.

This study has some limitations due to its retrospective, single-centre design. The relatively small sample size limited the scope for multivariate analysis, and therefore, the findings should be interpreted as exploratory and hypothesis-generating. As this was a retrospective observational study, causal relationships between frailty and outcomes cannot be inferred. We need to note that the CFS is a descriptive assessment and may be subject to observer bias or inter-observer variability. Nevertheless, the results provide useful insight into the correlation between frailty and outcomes in patients receiving NIV, which will help in clinical decision-making.

## Conclusions

This study highlights the importance of frailty, as measured by the CFS, in predicting short-term mortality among patients treated with NIV for acute type 2 respiratory failure. Patients with higher frailty scores experienced significantly worse outcomes, independent of age, highlighting the utility of frailty assessment in guiding treatment decisions and discussions about prognosis regarding the use of NIV in an acute setting. While our data did not demonstrate a significant association between frailty and short-term readmission, the observed trends suggest that frailty may still influence rehospitalisation risk.

Incorporating frailty assessment into routine practice may help clinicians stratify risk more accurately, tailor management strategies, and inform advance care planning for this vulnerable patient population. Future prospective multicentre studies are warranted to validate these findings and to further explore the role of frailty in shaping long-term outcomes in patients receiving acute NIV.
